# Neuronal-glial Interactions Define the Role of Nitric Oxide in Neural Functional Processes

**DOI:** 10.2174/157015912804143522

**Published:** 2012-12

**Authors:** Antonio Contestabile, Barbara Monti, Elisabetta Polazzi

**Affiliations:** Department of Pharmacy and Biotechnology, University of Bologna, Italy

**Keywords:** Nitric oxide, Nitric oxide synthases, Neurons, Astrocytes, Microglia.

## Abstract

Nitric oxide (NO) is a versatile cellular messenger performing a variety of physiologic and pathologic actions in most tissues. It is particularly important in the nervous system, where it is involved in multiple functions, as well as in neuropathology, when produced in excess. Several of these functions are based on interactions between NO produced by neurons and NO produced by glial cells, mainly astrocytes and microglia. The present paper briefly reviews some of these interactions, in particular those involved in metabolic regulation, control of cerebral blood flow, axonogenesis, synaptic function and neurogenesis. Aim of the paper is mainly to underline the physiologic aspects of these interactions rather than the pathologic ones.

## INTRODUCTION

Around 25 years ago endothelium-derived relaxing factor (EDRF) was identified as nitric oxide (NO), produced by the endothelial component of blood vessels and resulting in strong relaxation of the smooth muscles of the same structures [1-4]. First demonstration for a role of EDFR in brain function almost immediately followed when Garthwaite and co-workers showed that it was released by cerebellar neurons upon activation of the excitatory NMDA glutamate receptor [5]. It is noteworthy that, in this early report, the authors pointed at neuron-glia interactions as a key mechanism sub-serving the novel discovered function, stating that “….it may link activation of postsynaptic NMDA receptors to functional modifications in neighboring pre-synaptic terminals and glial cells”. The subsequent identification of NO as the actual messenger and the disclosure of the GMP system as the main transduction system of message inside the cell [6-8] opened the way to an impressive amount of research, which has produced more than 20,000 published papers listed in PubMed data bank. Since the beginning of research, the dual nature of NO in the brain was clearly identified with respect to its physiologic or pathologic roles, thus contributing to the attribution of the name of Janus-faced molecule [9-13]. Physiologic actions of NO are related to a vast array of neural function from neurogenesis and development to synaptic plasticity, learning and memory, while pathologic actions have been related to excessive production of the messenger in conditions of brain insults and neuroinflammation. While an intuitive explanation is that low concentrations of NO mediate physiologic actions and high concentrations pathologic actions, this may be a too easy way to address functions of a molecule so unstable, reactive and short-lived as NO, not forgetting that to estimate actual concentrations of NO has been a very difficult and still controversial task [14]. While concentrations in the µM range have been often evaluated to be physiologic in several tissues, including the nervous system, recent evidences resulting from application of multiple methods suggest much lower physiologic concentration in the low nanomolar range or even fractions of it [14]. In this complex picture, a peculiar additional complication offered by the nervous tissue is related to the multiple sources of brain NO, from neurons and from different types of glia, as well as from their interactions. Some of these mechanisms and of the related interactions will be considered in the present paper and their relevance to physiology, as well as in some instances also to pathology, will be discussed.

Nitric oxide is produced through a reaction catalyzed by enzymes called Nitric Oxide Synthases (NOSs) through a well studied reaction, in which a molecule of arginine is converted to citrulline releasing a molecule of NO and producing a molecule of water through NADPH oxidation, with a two step reaction in which N-omega-hydroxyl-L-arginine is an intermediate product [15]. Details on the enzyme structure, role of co-factors and reaction mechanism have been reviewed elsewhere [16]. In addition to NO, NOSs can produce, under some reaction conditions, both superoxide anion and hydrogen peroxide [17, 18]. Among the several reactive oxygen species (ROS) produced as side effect of oxidative metabolism, superoxide anion not scavenged by superoxide dismutase (SOD) is a favored reactant for NO, resulting in formation of peroxynitrite ion, which is an agent of relatively slow oxidative damage to most biological molecules, including proteins nucleic acids and lipids [19, 20]. The biochemistry and the physiopathological roles of peroxynitrite have been recently reviewed [21, 22]. Peroxynitrite is a relatively unstable ion and its reaction with protein tyrosine groups produces nitrotyrosines, a more stable footprint of nitrosative stress. Peroxynitrite is the most relevant source of NO-derived damage to biological molecules through oxidative and nitrosative stress, bringing to extensive cellular injury and possibly ending in cell death when produced in high concentration [20]. When produced at more physiologic concentration, however, peroxynitrite is an important modulator of many signaling pathways, such as kinase cascades, that play fundamental roles in cell physiology [20]. NO and peroxynitrite toxicity is lower compared to many ROS, such as superoxide anion, which it is able to scavenge or whose formation counteracts, or the very toxic hydroxyl radical, mainly produced by hydrogen peroxide through the Fenton reaction. Thus, rather paradoxically, NO may have antioxidant properties by counteracting formation of more toxic ROS species and also from formation of S-nitrosoglutathione (GSNO), which has a much higher antioxidant potency than GSH, and from its ability to block the lipid peroxidation chain reaction by forming nitrosylated lipids [23, 24].

NO is synthesized in the brain by three isoforms of NOSs: two are constitutive and calcium-dependent, the neuronal (nNOS or NOS1) and the endothelial (eNOS or NOS3) isoforms, and one is inducible and non-calcium-dependent (iNOS or NOS2). The two constitutive NOS isoforms, and in particular nNOS, have been implicated in the synaptic functions of NO, such as participation in synaptic plasticity and regulation of neurotransmitter release [25-27]. Neuronal NOS is the most abundant isoform in nervous parenchyma [27] and is structurally and functionally linked to the NMDA receptor in neurons through PDZ domains of postsynaptic density protein-95 (PSD-95) [28]. Several types of post-translational modifications, preeminently through phosphorylation, of nNOS that regulate its activity have been described [27]. Main cellular localization of eNOS in brain is obviously in vessel endothelium [29], where the enzyme is prevailingly linked to membrane invaginations in association with caveolin together with other proteins [30]. Reports localizing eNOS in neurons and astrocytes are more controversial [28, 30, 31]. The inducible isoform, i.e. iNOS, has a prevailingly glial localization and has been usually considered as almost undetectable in normal conditions, but strongly expressed by gene induction following insults, such as ischemia and neuroinflammation [32, 33]. Microglia, the resident immune cells of the brain are thought to massively contribute to NO production in these conditions. It has been, however, recently proposed that iNOS may be expressed by astrocytes to modulate normal neuronal function and in particular synaptic release of neurotransmitters [34]. Expression of NOSs from oligodendrocytes is also controversial: expression of both eNOS and iNOS has been reported under particular conditions in culture, while no conclusive evidence does exist regarding *in vivo* situation [35]. The whole picture of sites of NO production and of NO action in the brain is remarkably intriguing and undoubtedly neuron-glia interactions play a primary role, which may become overwhelming in pathologic conditions, but is also essential in physiologic conditions to define neural activity.

## NITRIC OXIDE-MEDIATED METABOLIC AND FUNCTIONAL INTERACTIONS IN ASTROCYTES

Astrocytes, being strategically located among neurons and between neurons and blood vessels, play critical roles in brain function. In particular, through their vascular end-feet processes, they envelop vessel walls and readily absorb nutrients, in particular glucose, from blood flow [36]. Furthermore, astrocytic processes, surrounding synaptic contacts and clefts, are essential for several steps of synaptic transmission, from the production of neurotransmitters and their precursors to their clearance from synaptic and extrasynaptic spaces [37]. Due to their central role in brain energy metabolism, the NO-dependent regulation of energy metabolism in astrocytes is a key element in the NO-dependent neuronal-glial interactions modulating brain function. This key role is further enhanced by the fact that astrocytes are the only brain cells that accumulate glycogen from glucose and thus they can act as energy supplying elements in conditions of metabolic stress and deficient energy production. A NO-dependent reversible inhibition of cytochrome c oxidase modulating mitochondrial respiration has been reported in several cellular and subcellular systems, including isolated mitochondria, synaptosomes, macrophages, neurons and astrocytes [38-42]. Inhibition is due to NO competition with oxygen, but prolonged mitochondrial exposure to NO results in persistent inhibition of cytochrome c oxydase through formation of peroxynitrite [43-44]. Neurons are highly vulnerable to this type of mitochondrial inhibition, while astrocytes are much more resistant and they respond by activating mechanisms improving their survival and, as a consequence, also neuronal survival. Most important, among these mechanisms there are: compensative increase of glucose uptake by astrocytes through increased expression of the GLUT1 and GLUT3 transporters mediated by NO-dependent activation of 5’-AMP-activated protein kinase; maintenance of high reducing potency through efficient regeneration of reduced glutathione (GSH) due to stimulation of glucose utilization through the pentose-phosphate pathway; enhancement of glycolytic pathway, in response to inhibition of mitochondrial respiration, in such a way to maintain high levels of ATP production [43-48].

Another important mechanism of NO-mediated interactions between neurons and astrocytes is related to various steps of synaptic transmission. As pointed out in a recent review [27], a distinctive feature of NO compared with other synaptic signaling molecules is its free diffusion through aqueous and lipid environments, thus making it able to act both at pre- and postsynaptic level, hence coordinating responses at both sides of a synapse. Astrocyte-derived NO undoubtedly shares the same ability to influence activity of adjoining synapse, or better synapses, considering its diffusion coefficient and traveling speed [27]. Synaptic action of NO should be first of all considered for its metabolic significance. Astrocytes efficiently internalize the only NO precursor, arginine, through the cationic amino acid transporters, and release it to their processes surrounding the synaptic cleft, where arginine can be taken on by neurons and converted to NO. On the other hand, glutamate released by excitatory terminals can enhance arginine release from astrocyte processes [49], thus ensuring higher level of substrate availability to the synaptic terminal and to the post-synaptic neuron. In this way, an efficient coupling is established between astrocyte arginine metabolism and neuronal NO synthesis. Interestingly, this mechanism is neuronal activity-dependent and, thus, potentially able to influence synaptic plasticity, as well as related mechanisms of learning and memory. Other interactions between astrocyte-derived NO and synaptic activity have been described in mixed cultures of hippocampal neurons and astrocytes with opposite effects: neuronal network exhibits synchronous calcium oscillations due to spontaneous glutamate release from synaptic terminals [50], while astrocytes, responding to co-released ATP through a specific receptor, produce NO that has a depressive effect on further synaptic activity and calcium oscillations.

The identification of physiologic roles in synaptic function of NO derived from astrocytic iNOS is rendered conceptually difficult by the prevailing idea that this enzyme is only induced through gene expression in conditions of un-physiologic stimulation and pathologic brain insult [33, 34]. However, evidence has been reported in some studies for a constitutive NO expression at baseline levels in astrocytes [51-54] and it has been proposed that the consequent NO production at the astrocytic-neuronal interface modulates synaptic activity [55]. Selective iNOS inhibitors were able to decrease NO signals from brain slices even in conditions of protein blockade and RT-PCR analysis revealed the presence of iNOS mRNA in the tissue [51]. A similar analysis demonstrated that iNOS mRNA was about 20% of nNOS mRNA in rat ventral medulla and iNOS inhibition *in vivo* was able to affect sympathetic tone [52]. Low levels of iNOS expression in some astrocytes, and in some microglial cells as well, were revealed through immunohistochemistry in the normal striatum of rats and the level of expression was greatly enhanced by beta-amyloid injection [56]. In mouse cortex non calcium-dependent NOS activity attributable to iNOS contributes with a 10% fraction of total NOS activity [53, 54]. Furthermore, both cortical and amygdala slices demonstrated the ability to produce NO, in a iNOS-dependent inhibitory setting, in the presence of protein synthesis inhibitors [53, 57]. On the basis of these studies, as well as of other observations derived from the literature [58-60], the idea that iNOS from astrocytes contributes to physiologic synaptic function [55] has gained momentum even if it will require further support before gaining a generalized consensus among experts of the field.

If it is to be ascertained whether and to what extent NO derived from astrocytes can influence synaptic function in neurons, there is little doubt on neuronal-derived NO influence upon astrocyte function. This is mainly due to the fact that astrocytes are highly responsive to NO-mediated cGMP production [61-64]. Some results suggest that the cGMP rise, readily occurring in astrocytes in response to NO stimulation, results in an increase of calcium concentration, in GFAP expression, the main intermediate filament of astrocytes, and in cytoskeleton remodeling [65-68]. Mechanisms of this kind may be involved in the regulation of motility of radial glial cells during development, as described in the optic tectum of Xenopus tadpoles [69] In this model, *in vivo* observations demonstrated that extension and retraction of filopodial processes from radial glia was modulated by visual stimulation through NO signaling downstream NMDA receptor activation [69]. These interactions may be at the basis of the role ascribed to NO in the refinement of retino-tectal and retino-geniculate projections [70-72] and in the regulation of the wiring of the insect nervous system [73]. NO-mediated interactions are not restricted to mechanisms of axon migration and wiring, but they are also involved in developmental migration of neuroblasts and neuronal precursors [74-76]. Inflammatory induction of iNOS has been described in astrocytes upon administration of inflammogen molecules, such as the bacterial endotoxin lipopolysaccharide (LPS) or cytokines, and in animal models of neuroimmune diseases [77-81]. Interestingly, this induction is modulated by physiologic mechanisms, such as neurotransmitters, noradrenaline for instance, and regulation of transcriptional machinery through sumoylation [82-84]. A comprehensive review of signals that induce iNOS in astrocytes has been published recently [85].

Another important functional correlate implicating astrocytes and NO is related to the fact that these cells are in close morphologic and functional correlation with neurons and blood vessels. Thus astrocytes are strategically located at midway between two important sources of NO, from neurons and from endothelial cells, and it is well established that they are essential actors in the regulation of cerebral blood flow [86, 87]. This strategic location of astrocytes has contributed to develop the concept of neurovascular unit, as a group of neurons and associated astrocytes functionally coupled to endothelium and smooth muscular elements of small blood vessels, regulating blood supply to the parenchyma [88, 89]. During neuronal stimulation, nNOS activated by calcium flowing through NMDA receptors produces NO, which can diffuse and directly affect vasodilation by activating guanylate cyclase in smooth muscle cells. In addition, NO produced in this way may modulate astrocyte signaling leading to activation of additional vasodilatory signals [86, 87]. Pharmacologic approaches to dissect the contribution of these different mechanisms suggest that, in addition to direct effect of neuronal-derived NO on smooth muscle cells of blood vessels, interactions between NO and astrocytic arachidonic acid pathway are involved in the vasodilatory effect and that further NO may derive from endothelial cells stimulated mechanically by blood flow or neurohumorally [87]. The vasodilatory effect of NO exerted through arachidonic acid pathways in astrocytes has multiple and still not entirely understood targets. One effect is through stimulation of cyclooxygenase 1 in astrocytes, which leads to production of vasodilatory prostaglandins [90]. A second way is through regulation of the balance between inhibition of the production of the vasoconstrictor metabolite of arachidonic acid, 20-hydroxyeicosatetranoic acid, and of the production of the vasodilator metabolite, epoxyeicosatrienoic acid [91, 92]. The shift of the balance between the inhibition of the production of the two metabolites may explain why under certain circumstances, such as light-induced response in the retina, NO may result vasoconstrincting instead of vasodilating [92]. Another obvious element in determining the final effect of neuronal-derived NO on blood flow regulated through direct action or through modulation of astrocytes is the different NO-producing capacity in different brain regions. In the cerebellum, for instance, NOS activity is tenfold higher than in the somatosensory cortex [93] and several other brain regions have intermediate values 

## NITRIC OXIDE IN THE CROSS-TALK BETWEEN MICROGLIA AND NEURONS

The communication between the immune system and central nervous system (CNS) is crucial for suitable physiologic, immunologic and behavioral response to normal aging, infections and injuries. Microglial cells, constituting approximately 10–12% of brain cell population, are the unique immune population of the CNS and represent the resident macrophages in the brain. These cells are largely present in the grey matter, with higher density in the hippocampus, hypothalamus, basal ganglia and substantia nigra [94-96]. In the healthy adult brain, microglia are normally in a quiescent or “resting” state and are morphologically characterized by a small soma and ramified processes [97]. These cells, however, are not dormant, since they possess highly motile processes that scan the CNS microenvironment [98, 99]. Minor alterations in brain homeostasis alert microglia that provide the first line of defense. During trauma, ischemia, infection and various neurodegenerative diseases, microglia change morphology, gene expression and perform a variety of functions essential to the immune and inflammatory response of the CNS. These functions include the release of pro- and anti-inflammatory molecules, the presentation of antigens to T cells and the phagocytosis of tissue debris, damaged cells and microorganisms [reviewed in 97, 100-102]. While microglial activation is necessary and beneficial in response to injury or disease, prolonged or excessive activation may have deleterious effects on brain functions. For this reason, in the healthy brain, microglia reactivity is actively modulated by neurons. Several molecules orchestrate the cross-talk between neurons and microglial cells in the healthy, as well as in the damaged brain and, among them, NO has a primary role. As summarized in the first paragraph of the present paper, nNOS is the primary source of neuronal-derived NO, while iNOS induced by activation is the primary source of microglia-derived NO.

Several studies have been made to elucidate the role of microglial-derived NO on neuronal survival, mainly focusing on damage-exacerbating effects of excessive NO production by activated microglial cells. For example, it has been shown that high levels of NO derived from microglia stimulated with LPS induced neuronal death by inhibition of neuronal respiration. NO inhibition of neuronal respiration caused, in turn, neuronal depolarization and glutamate release, followed by excitotoxicity *via *the NMDA receptor [103-106]. Accordingly, NMDA receptor-mediated toxicity towards cerebellar granule neurons was potentiated by co-culture with immunostimulated microglia or by co-exposure to an NO donor [107]. Additional evidences on the neurotoxic role of excessive NO production from activated microglia comes from studies demonstrating exacerbation of damage due to excitotoxic-like conditions, such as glucose deprivation and hypoxia [108-110]. Moreover it has been demonstrated that nitric oxide caused neuronal death not only *via *NMDA receptors, but it could also act directly upon immature neurons, through a not fully defined mechanism [111]. Recently, Graber *et al*., [112] further supported NO production as an important mediator in microglia-induced neuron death. Damaging actions of NO, mostly mediated by microglia activation, have been extensively considered in recent reviews [113, 114]. These data, mostly obtained in *in vitro* systems, suggest that inflammatory conditions lead microglia to assume a neurodestructive phenotype and that induction of iNOS with NO overproduction plays a central role in the neurodegenerative mechanism. Other *in vitro* results help to better understand the complex relationships between microglia and neurons, in which NO plays a role. By using neuron-conditioned media, we demonstrated that differentiated neurons release in the medium substances able to control microglia activation and to induce apoptosis of immunostimulated microglia [115] and subsequent evidence suggested a role for NO in this process. Indeed, co-cultures of cerebellar granule neurons and microglia or exposure of microglia to a medium previously conditioned by the same neurons strongly induced iNOS expression and NO production by LPS-activated microglia [116]. Prolonging to 72 hours the exposure of activated microglia to neuron-conditioned medium induced their apoptosis, an effect interpreted as a safety mechanism to avoid excessive and dangerous inflammatory response [116]. The existence of other NO production auto-regulated mechanisms through iNOS, have been suggested by experiments on a microglia-derived cell line, BV-2 cells, in which NO negatively regulated LPS-induced iNOS expression to avoid excessive NO production [117]. 

*In vitro* demonstrations may be difficult to be translated *in vivo*, since NO levels actually present *in vivo* are not easily determined and several natural scavengers, such as hemoglobin of circulating red blood cells and other biological molecules able to react with NO, may contribute to render its concentration very variable and rather unpredictable. However, some interesting results have been obtained on neurogenesis, on its regulation by NO and on the dependence of this regulation by nNOS or iNOS derived NO. In general terms, the fact that NO produced in brain physiologic conditions, mainly through nNOS, has an antiproliferative action on neuronal precursors is well established. This has been demonstrated both in developmental and in adult neurogenesis in the two brain areas, the hippocampal subgranular zone of the dentate gyrus and the subventricular zone of the lateral ventricles, that maintain lifelong neurogenetic activity [118-124]. However, multiple experimental evidences suggest that NO may instead stimulate neurogenesis in the hippocampal dentate gyrus following different types of damage and that activated microglia is primarily responsible for the NO overproduction under these conditions [125-130]. This iNOS-related microglia action can be interpreted as a mechanism helping the recovery of neural function after damage. This dual role of NO, related to the source of the messenger molecule and to the presence of physiologic (nNOS prevailingly active) or pathologic (iNOS primarily active) conditions, allows to further appreciate the complicated interplay between NO production and the physiopathologic state of neurons and glia, in particular microglia. In this complex interplay, we have recently disclosed a novel mechanism through which microglia regulates nNOS expression in neurons through proteolysis, as illustrated in the next paragraph.

## CONCLUDING REMARKS

Neuron-glia interactions are of foremost importance in the regulation of neural function, both regarding metabolic aspects and communication flow in the neuronal network. Nitric oxide, an important messenger molecule in most tissue, is involved in many of these functions. It is, therefore, interesting to consider the role of NO in these functions, as this molecule is produced in the nervous tissue by neurons, astrocytes and microglia. The fact that glial-derived NO is neurotoxic, when produced in excess, has often focused the interest of investigators on the neuropathologic side of NO actions. However, regulated production of NO from both neurons and glia must be primarily appreciated for its physiologic regulation of many neural functions both during development and in adult. By adopting a well known metaphor, this may bring to the suggestion that, dealing with NO, the “good” face of Janus must be considered at least as much important as the “bad” face. In the present paper, we have tried to highlight the most important among the many NO-dependent functional interactions between neurons and glia, in order to provide researchers with a background view, able to stimulate future investigation. 

## References

[1] Ignarro LJ, Buga GM, Wood KS, Byrns RE, Chaudhuri G (1987). Endothelium-derived relaxing factor produced and released from artery and vein is nitric oxide. Proc. Natl. Acad. Sci. USA.

[2] Palmer RM, Ferrige AG, Moncada S (1987). Nitric oxide release accounts for the biological activity of endothelium-deriverelaxing factor. Nature.

[3] Ignarro LJ (2002). Nitric oxide in the regulation of vascular function: an historical overview. J. Card. Surg.

[4] Murad F (2006). Nitric oxide and cyclic GMP in cell signaling and drug development. N. Engl. J. Med.

[5] Garthwaite J, Charles SL, Chess-Williams R (1988). Endothelium-derived relaxing factor release on activation of NMDA receptors suggests role as intercellular messenger in the brain. Nature.

[6] Bredt DS, Snyder SH (1989). Nitric oxide mediates glutamate-linked enhancement off cGMP levels in the cerebellum. Proc. Natl. Acad. Sci. USA.

[7] Garthwaite J, Garthwaite G, Palmer RM, Moncada S (1989). NMDA receptor activation induces nitric oxide synthesis from arginine in rat brain slices. Eur. J. Pharmacol.

[8] Knowles RG, Palacios M, Palmer RM, Moncada S (1989). Formation of nitric oxide from L-arginine in the central nervous system: a transduction mechanism for stimulation of the soluble guanylate cyclase. Proc. Natl. Acad. Sci. USA.

[9] Snyder SH (1993). Janus faces of nitric oxide. Nature.

[10] Calabrese V, Cornelius C, Rizzarelli E, Owen JB, Dinkova-Kostova T, Butterfield DA (2009). Nitric oxide in cell survival: a janus molecule. Antiox. Redox Signal.

[11] Brown GC (2010). Nitric oxide and neuronal death. Nitric Oxide.

[12] Steinert JR, Chernova T, Forsythe ID (2010). Nitric oxide in 
brain function, dysfunction and dementia. Neuroscientist.

[13] Nakamura T, Lipton SA (2011). Redox modulation by S-nitrosylation contributes to protein misfolding, mitochondrial dynamics, and neuronal synaptic damage in neurodegenerative diseases. Cell Death Differ.

[14] Hall CN, Garthwaite J (2009). What is the real physiological NO concentration *in vivo*?. Nitric Oxide.

[15] Stuehr DJ, Kwon NS, Nathan CF, Griffith OW, Feldman PL, Wiseman J (1991). N omega-hydroxyl-L-arginine is an intermediate in the biosynthesis of nitric oxide from L-arginine. J. Biol. Chem.

[16] Contestabile A, Monti B, Contestabile A, Ciani E (2003). Brain nitric oxide and its dual role in neurodegeneration/neuroprotection: understanding molecular mechanisms to devise drug approaches. Curr. Med. Chem.

[17] Heinzel B, John M, Klatt P, Bohme E, Mayer B (1992). Ca^2+^/calmodulin-dependent formation of hydrogen peroxide by brain nitric oxide synthase. Biochem. J.

[18] Pou S, Pou WS, Bredt DS, Snyder SH, Rosen GM (1992). Generation of superoxide by purified brain nitric oxide synthase. J. Biol. Chem.

[19] Beckman JS, Koppenol WH (1996). Nitric oxide, superoxide and peroxynitrite: the good, the bad and the ugly. Am. J. Physiol.

[20] Pacher P, Beckman JS, Liaudet L (2007). Nitric oxide and peroxynitrite in health and disease. Physiol. Rev.

[21] Koppenol WH (2001). 100 years of peroxynitrite chemistry and 11 years of peroxynitrite biochemistry. Redox. Rep.

[22] Radi R (2009). Peroxynitrite and reactive nitrogen species: the contribution of ABB in two decades of research. Arch. Biochem. Biophys.

[23] Chiueh CC (1999). Neuroprotective properties of nitric oxide. Ann. N.Y. Acad. Sci.

[24] Ciueh CC, Rauhala P (1999). The redox pathway of S-nitrosoglutathione, glutathione and nitric oxide in cell to neuron communications. Free Rad. Res.

[25] Prast H, Philippu A (2001). Nitric oxide as modulator of neuronal function. Prog. Neurobiol.

[26] Micheva KD, Buchanan J, Holz RW, Smith SJ (2003). Retrograde regulation of synaptic vesicle endocytosis and recycling. Nat. Neurosci.

[27] Garthwaite J (2008). Concepts of neural nitric oxide-mediated transmission. Eur. J. Neurosci.

[28] Brenman JE, Chao DS, Gee SH, McGee AW, Craven SE, Santillano DR, Wu Z, Huang F, Xia H, Peters MF, Froehner SC, Bredt DS (1996). Interaction of nitric oxide synthase with the postsynaptic density protein PSD-95 and alpha1-syntrophin mediated by PDZ domains. Cell.

[29] Blackshaw S, Eliasson MJ, Sawa A, Watkins CC, Krug D, Gupta A, Arai T, Ferrante RJ, Snyder SH (2003). Species, strain and developmental variations in hippocampal neuronal and endothelial nitric oxide synthase clarify discrepancies in nitric oxide-dependent synaptic plasticity. Neuroscience.

[30] Cirino G, Fiorucci S, Sessa WC (2003). Endothelial nitric oxide synthase: the Cinderella of inflammation?. Trends Pharmacol. Sci.

[31] O’Dell TJ, Huang PL, Dawson TM, Dinerman JL, Snyder SH, Kandel ER, Fishman MC (1994). Endothelial NOS and the blockade of LTP by NOS inhibitors in mice lacking neuronal NOS. Science.

[32] Lin LH, Taktakishvili O, Talman WT (2007). Identification and localization of cell types that express endothelial and neuronal nitric oxide synthase in the rat nucleus tractus solitarii. Brain Res.

[33] Aktan F (2004). iNOS-mediated nitric oxide production and its regulation. Life sci.

[34] Saha RN, Pahan K (2006). Regulation of inducible nitric oxide synthase gene in glial cells. Antioxid. Redox Signal.

[35] Boullerne AI, Benjamins JA (2006). Nitric oxide synthase expression and nitric oxide toxicity in oligodendrocytes. Antiox. Redox Signal.

[36] Morgello S, Uson RR, Schwartz EJ, Haber RS (1995). The human blood-brain barrier glucose transporter (GLUT1) is a glucose transporter of gray matter astrocytes. Glia.

[37] Blumcke I, Eggli P, Celio MR (1995). Relationship between astrocytic processes and “perineuronal nets” in rat neocortex. Glia.

[38] Brown GC (1995). Nitric oxide regulates mitochondrial respiration and cell functions by inhibiting cytochrome oxidase. FEBS Lett.

[39] Brown GC, Cooper CE (1994). Nanomolar concentrations of nitric oxide reversibly inhibit synaptosomal respiration by competing with oxygen at cytochrome oxidase. FEBS Lett.

[40] Cleeter MWJ, Cooper JM, Darley-Usmar VM, Moncada S, Schapira AH (1994). Reversible inhibition of cytochrome c oxidase, the terminal enzyme of the mitochondrial respiratory chain, by nitric oxide. Implications for neurodegenerative diseases. FEBS Lett.

[41] Brorson JR, Schumacker PT, Zhang H (1999). Nitric oxide acutely inhibits neuronal energy metabolism. J. Neurosci.

[42] Szabó C, Day BJ, Salzman AL (1996). Evaluation of the relative contribution of nitric oxide and peroxynitrite to the suppression of mitochondrial respiration in immunostimulated macrophages using a manganese mesoprophyrin superoxide dismutase mimetic and peroxynitrite scavenger. FEBS Lett.

[43] Bolaños JP, Peuchen S, Heales SJR, Land JM, Clark JB (1994). Nitric oxide-mediated inhibition of the mitochondrial respiratory chain in cultured astrocytes. J. Neurochem.

[44] Sharpe MA, Cooper CE (1998). Interaction of peroxynitrite with mitochondrial cytochrome oxidase. J. Biol. Chem.

[45] Almeida A, Cidad P, Delgado-Esteban M, Fernandez E, Garcia-Nogales P, Bolanos JP (2005). Inhibition of mitochondrial respiration by nitric oxide:its role in glucose metabolism and neuroprotection. J. Neurosci. Res.

[46] Bolanos JP, Almeida A (2006). Modulation of astroglial energy metabolism by nitric oxide. Antiox. Redox Signal.

[47] Moncada S, Bolanos JP (2006). Nitric oxide, cell bioenergetics and neurodegeneration. J. Neurochem.

[48] Bolanos JP, Delgado-Esteban M, Herrero-Mendez A, Fernandez-Fernandez S, Almeida A (2008). Regulation of glycolysis and penose-phosphate pathway by nitric oxide: impact on neuronal survival. Biochim. Biophys. Acta.

[49] Gensert JM, Ratan RR (2006). The metabolic coupling of arginine metabolism to nitric oxide generation by astrocytes. Antiox. Redox Signal.

[50] Mehta B, Begum G, Joshi NB, Joshi PG (2008). Nitric oxide mediated modulation of synaptic activity by astrocytic P2Y receptors. J. Gen. Physiol.

[51] Starkey SJ, Grant AL, Hagan RM (2001). A rapid and transient synthesis of nitroc oxide (NO) by a constitutively expressed type II NO synthase in the guinea-pig suprachiasmatic nucleus. Br. J. Pharmacol.

[52] Chan SH, Wang LL, Chan JY (2003). Differential engagements of glutamate and GABA receptors in cardiovascular actions of endogenous nNOS or iNOS at rostral ventrolateral medulla of rats. Br. J. Pharmacol.

[53] Buskila Y, Farkash S, Hershfinkel M, Amitai Y (2005). Rapid and reactive nitric oxide production by astrocytes in mouse neocortical slices. Glia.

[54] Buskila Y, Amitai Y (2010). Astrocytic iNOS-dependent enhancement of synaptic release in mouse neocortex. J. Neurophysiol.

[55] Amitai Y (2010). Physiologic role for “inducible” nitric oxide synthase: a new form of astrocytic-neuronal interface. Glia.

[56] Weldon DT, Rogers SD, Ghilardi JR, Finke MP, Cleary JP, O’Hare E, Esler WP, Maggio JE, Mantyh PW (1998). Fibrillar beta-amyloid induces microglial phagocytosis, expression of inducible nitric oxide synthase, and loss of a select population of neurons in the rat CNS *in vivo*. J. Neurosci.

[57] Abu-Ghanem Y, Cohen H, Buskila Y, Grauer E, Amitai Y (2008). Enhanced stress reactivity in nitric oxide synthase type 2 mutant mice: Findings in support of astrocytic nitrosative modulation of behavior. Neuroscience.

[58] Ikeda H, Kusudo K, Murase K (2006). Nitric oxide-dependent long-term potentiation revealed by real-time imaging of nitric oxide production and neuronal excitation in the dorsal horn of rat spinal cord slices. Eur. J. Neurosci.

[59] Ikeda H, Murase K (2004). Glial nitric oxide-mediated long-term presynaptic facilitation revealed by optical imaging in rat spinal dorsal horn. J. Neurosci.

[60] Buskila Y, Abu-Ghanem Y, levi Y, Moran A, Grauer E, Amitai Y (2007). Enhanced astrocytic nitric oxide production and neuronal modifications in the neocortex of a NOS2 mutant mouse. PLoS One.

[61] de Vente J, Bol JGJM, Berkelmans HS, Schipper J, Steinbusch HW (1990). Immunocytochemistry of cGMP in the cerebellum of the immature, adult, and aged rat: the involvement of nitric oxide. A micropharmacological study. Eur. J. Neurosci.

[62] de Vente J, Hopkins DA, Markerink-Van Ittersum M, Emson PC, Schmidt HH, Steinbusch HW (1998). Distribution of nitric oxide synthase and nitric oxide-receptive, cyclic GMP-producing structures in the rat brain. Neuroscience.

[63] Southam E, Garthwaite J (1993). The nitric oxide-cyclic GMP signalling pathway in rat brain. Neuropharmacology.

[64] Southam E, Morris R, Garthwaite J (1992). Sources and targets of nitric oxide in rat cerebellum. Neurosci. Lett.

[65] Matyash V, Filippov V, Mohrhagen K, Kettenmann H (2001). Nitric oxide signals parallel fiber activity to Bergmann glial cells in the mouse cerebellar slice. Mol. Cell. Neurosci.

[66] Willmott NJ, Wong K, Strong AJ (2000). A fundamental role for the nitric oxide-G-kinase signaling pathway in mediating intercellular Ca(2+) waves in glia. J. Neurosci.

[67] Brahmachari S, Fung YK, Pahan K (2006). Induction of glial fibrillary acidic protein expression in astrocytes by nitric oxide. J. Neurosci.

[68] Boran MS, Garcia A (2007). The cyclic GMP-protein kinase G pathway regulates cytoskeleton dynamics and motility in astrocytes. J. Neurochem.

[69] Tremblay M, Fugère V, Tsui J, Schohl A, Tavakoli A, Travençolo BA, Costa Lda F, Ruthazer ES (2009). Regulation of radial glial motility by visual experience. J. Neurosci.

[70] Wu HH, Williams CV, McLoon SC (1994). Involvement of nitric oxide in the elimination of a transient retinotectal projection in development. Science.

[71] Cramer KS, Angelucci A, Hahm JO, Bogdanov MB,  
Sur M (1996). A role for nitric oxide in the development of the ferret retinogeniculate projection. J. Neurosci.

[72] Leamey CA, Ho-Pao CL, Sur M (2001). Disruption of retinogeniculate pattern formation by inhibition of soluble guanylyl cyclase. J. Neurosci.

[73] Bicker G (2005). STOP and GO with NO: nitric oxide as a regulator of cell motility in simple brains. BioEssays.

[74] Tanaka M, Yoshida S, Yano M, Hanaoka F (1994). Roles of endogenous nitric oxide in cerebellar cortical development in slice cultures. Neuroreport.

[75] Enikolopov G, Baneriji J, Kuzin B (1999). Nitric oxide and Drosophila development. Cell Death Differ.

[76] Tegenge MA, Rockel TD, Fritsche E, Bicker G (2011). Nitric 
oxide stimulates human neural progenitor cell migration *via* 
cGMP-mediated signal transduction. Cell Mol Life Sci.

[77] Hewett SJ, Corbett JA, McDaniel ML, Choi DW (1993). Interferongamma and interleukin-1 beta induce nitric oxide formation from primary mouse astrocytes. Neurosci. Lett.

[78] Lee SC, Dickson DW, Liu W, Brosnan CF (1993). Induction of nitric oxide synthase activity in human astrocytes by interleukin-1 beta and interferon-gamma. J. Neuroimmunol.

[79] Simmons ML, Murphy S (1992). Induction of nitric oxide synthase in glial cells. J. Neurochem.

[80] Bagasra O, Michaels FH, Zheng YM, Bobroski LE, Spitsin SV, Fu ZF, Tawadros R, Koprowski H (1995). Activation of the inducible form of nitric oxide synthase in the brains of patients with multiple sclerosis. Proc. Natl. Acad. Sci. USA.

[81] Bo L, Dawson TM, Wesselingh S, Mork S, Choi S, Kong PA, Hanley D, Trapp BD (1994). Induction of nitric oxide synthase in demyelinating regions of multiple sclerosis brains. Ann. Neurol.

[82] Feinstein DL, Galea E, Reis DJ (1993). Norepinephrine suppresses inducible nitric oxide synthase activity in rat astroglial cultures. J. Neurochem.

[83] Feinstein DL (1998). Suppression of astroglial nitric oxide synthase expression by norepinephrine results from decreased NOS-2 promoter activity. J. Neurochem.

[84] Akar CA, Feinstein DL (2009). Modulation of inducible nitric oxide synthase expression by sumoylation. J. Neuroinflam.

[85] Saha RN, Pahan K (2006). Signals for the induction of nitric oxide synthase in astrocytes. Neurochem. Int.

[86] Koehler RC, Roman RJ, Haerder DR (2008). Astrocytes and the regulation of cerebral blood flow. Trends Neurosci.

[87] Atwell D, Buchan AM, Charpak S, Lauritzen M, MacVicar BA, Newman EA (2010). Glial and neuronal control of brain blood flow. Nature.

[88] Iadecola C (2004). Neurovascular regulation in the normal brain and in Alzheimer’s disease. Nat. Rev. Neurosci.

[89] Jakovcevic D, Harder DR (2007). Role of astrocytes in matching blood flow to neuronal activity. Curr. Topics Devel. Biol.

[90] Fujimoto Y, Uno E, Sakuma S (2004). Effect of reactive oxygen and nitrogen species on cyclooygenase-1 and -2 activities. Prost. Leukot. Essent. Fatty Acids.

[91] Sun CW, Falck JR, Okamoto H, Harder DR, Roman RJ (2000). Role of cGMP versus 20-HETE in the vasodilator response to nitric oxide in rat cerebral arteries. Am. J. Physiol. Heart Circ. Physiol.

[92] Metea MR, Newman EA (2006). Glial cells dilate and constrict blood vessels: a mechanism of neurovascular coupling. J. Neurosci.

[93] Cholet N, Bonvento G, Seylaz J (1996). Effect of neuronal NO synthase inhibition on the cerebral vasodilatory response to somatosensory stimulation. Brain Res.

[94] Block ML, Zecca L, Hong JS (2007). Microglia-mediated neurotoxicity: uncovering the molecular mechanisms. Nat. Rev. Neurosci.

[95] Lawson LJ, Perry VH, Dri P, Gordon S (1990). Heterogeneity in the distribution and morphology of microglia in the normal adult mouse brain. Neuroscience.

[96] Mittelbronn M, Dietz K, Schluesener HJ, Meyermann R (2001). Local distribution of microglia in the normal adult human central nervous system differs by up to one order of magnitude. Acta Neuropathol.

[97] Hanisch UK, Kettenmann H (2007). Microglia: active sensor and versatile effector cells in the normal and pathologic brain. Nat. Neurosci.

[98] Davalos D, Grutzendler J, Yang G, Kim JV, Zuo Y, Jung S, Littman DR, Dustin ML, Gan WB (2005). ATP mediates rapid microglial response to local brain injury *in vivo*. Nat. Neurosci.

[99] Nimmerjahn A, Kirchhoff F, Helmchen F (2005). Resting microglial cells are highly dynamic surveillants of brain parenchyma *in vivo*. Science.

[100] Kreutzberg GW (1996). Microglia: a sensor for pathological events in the CNS. Trends Neurosci.

[101] Ransohoff RM, Perry VH (2009). Microglial physiology: unique stimuli, specialized responses. Annu. Rev. Immunol.

[102] Graeber MB (2010). Changing face of microglia. Science.

[103] Boje KM, Arora PK (1992). Microglial-produced nitric oxide and reactive nitrogen oxides mediate neuronal cell death. Brain Res.

[104] McNaught KStP, Brown GC (1998). Nitric oxide causes glutamate release from brain synaptosomes following inhibition of mitochondrial function. J. Neurochem.

[105] Bal-Price A, Brown GC (2001). Inflammatory neurodegeneration mediated by nitric oxide from activated glia, inhibiting neuronal respiration, causing glutamate release and excitoxicity. J. Neurosci.

[106] Stewart VC, Heslegrave AJ, Brown GC, Clark JB, Heales SJ (2002). Nitric oxide-dependent damage to neuronal mitochondria involves the NMDA receptor. Eur. J. Neurosci.

[107] Kim WK, Ko KH (1998). Potentiation of N-methyl-d-aspartate-mediated neurotoxicity by immunostimulated murine microglia. J. Neurosci. Res.

[108] Mander P, Borutaite V, Moncada S, Brown GC (2005). Nitric oxide from inflammatory-activated glia synergize with hypoxia to induce neuronal death. J. Neurosci. Res.

[109] Moncada S, Bolanos JP (2006). Nitric oxide, cell bioenergetics and neurodegeneration. J. Neurochem.

[110] Kim WK, Seo DO, Choi JJ, Ko KH (1999). Immunostimulated glial cells potentiate glucose deprivation-induced death of cultured rat cerebellar granule cells. J. Neurotrauma.

[111] Golde S, Chandran S, Brown GC, Compston A (2002). Different pathways for iNOS-mediated toxicity *in vitro* dependent on neuronal maturation and NMDA receptor expression. J. Neurochem.

[112] Graber DJ, Snyder-Keller A, Lawrence DA, Turner JN (2012). Neurodegeneration by activated microglia across a nanofiltration membrane. J. Biochem. Mol. Toxicol.

[113] Brown GC (2007). Mechanisms of inflammatory neurodegeneration: iNOS and NADPH oxidase. Biochem. Soc. Trans.

[114] Brown GC, Neher JJ (2010). Inflammatory neurodegeneration and mechanisms of microglial killing of neurons. Mol. Neurobiol.

[115] Polazzi E, Contestabile A (2003). Neuron-conditioned media differentially affect the survival of activated or unstimulated microglia: evidence for neuronal control on apoptotic elimination of activated microglia. J. Neuropathol. Exp. Neurol.

[116] Polazzi E, Contestabile A (2006). Overactivation of LPS-stimulated microglial cells by co-cultured neurons or neuron-conditioned medium. J. Neuroimmunol.

[117] Yoshioka Y, Takeda N, Yamamuro A, Kasai A, Maeda S (2010). Nitric oxide inhibits lipopolysaccharide-induced inducible nitric oxide synthase expression and its own production through the cGMP signaling pathway in murine microglia BV-2 cells. J. Pharmacol. Sci.

[118] Peunova N, Scheinker V, Cline H, Enikolopov G (2001). Nitric oxide is an essential negative regulator of cell proliferation in Xenopus brain. J. Neurosci.

[119] Cheng A, Wang S, Cai J, Rao MS, Mattson MP (2003). Nitric oxide acts in a positive feedback loop with BDNF to regulate neural progenitor cell proliferation and differentiation in the mammalian brain. Dev. Biol.

[120] Packer MA, Stasiv Y, Benraiss A, Chmielnicki E, Grinberg H, Westphal H, Goldman SA, Enikolopov G (2003). Nitric oxide negatively regulates mammalian adult neurogenesis. Proc. Natl. Acad. Sci. USA.

[121] Moreno-Lopez B, Romero-Grimaldi C, Noval JA, Murillo-Carretero M, Matarredona ER, Estrada C (2004). Nitric oxide is a physiological inhibitor of neurogenesis in the adult mouse subventricular zone and olfactory bulb. J. Neurosci.

[122] Ciani E, Calvanese V, Crochemore C, Bartesaghi R, Contestabile A (2006). Proliferation of cerebellar precursor cells is negatively regulated by nitric oxide in newborn rat. J. Cell. Sci.

[123] Zhu XJ, Hua Y, Jiang J, Zhou OG, Luo CX, Han X, Lu YM, Zhu DY (2006). Neuronal nitric oxide synthase-derived nitric oxide inhibits neurogenesis in the adult dentate gyrus by down-regulating cyclic AMP response element binding protein phosphorylation. Neuroscience.

[124] Keilhoff G (2011). nNOS deficiency-induced cell proliferation depletes the neurogenic reserve. Neurosci. Lett.

[125] Zhang R, Zhang L, Zhang Z, Wang Y, Lu M, Lapointe  
M, C hopp M (2001). A nitric oxide donor induces neurogenesis and reduces functional deficits after stroke in rats. Ann. Neurol.

[126] Chen J, Zacharek A, Zhang C, Jiang H, Li Y, Roberts  
C, Lu M, Kapke A, Chopp M (2005). Endothelial nitric oxide 
synthase regulates brain derive neurotrophic factor expression 
and neurogenesis after stroke in mice. J. Neurosci.

[127] Zhu DY, Liu SH, Sun HS, Lu YM (2003). Expression of inducible nitric oxide synthase after focal cerebral ischemia stimulates neurogenesis in the adult rodent dentate gyrus. J. Neurosci.

[128] Cardenas A, Moro MA, Hurtado O, Leza JC, Lizasoain I (2005). Dual role of nitric oxide in adult neurogenesis. Brain Res. Rev.

[129] Luo CX, Zhu XJ, Zhou QG, Wang B, Wang W, Cai HH, Sun YJ, Hu M, Jiang J, Hua Y, Han X, Zhu DY (2007). Reduced neuronal nitric oxide synthase is involved in ischemia-induced hippocampal neurogenesis by up-regulating inducible nitric oxide synthase expression. J. Neurochem.

[130] Hua Y, Huang XY, Zhou L, Zhou QG, Hu Y, Luo CX, Li F, Zhu DY (2008). DETA/NONOate, a nitric oxide donor, produces antidepressant effects by promoting hippocampal neurogenesis. Psychopharmacology.

